# Added Value of Xpert MTB/RIF Ultra for Diagnosis of Pulmonary Tuberculosis in a Low-Prevalence Setting

**DOI:** 10.1128/JCM.01717-18

**Published:** 2019-01-30

**Authors:** Onya Opota, Fathiah Zakham, Jesica Mazza-Stalder, Laurent Nicod, Gilbert Greub, Katia Jaton

**Affiliations:** a Institute of Microbiology, University of Lausanne, University Hospital of Lausanne, Lausanne, Switzerland; b Pneumology Service, University Hospital of Lausanne, Lausanne, Switzerland; c Infectious Diseases Service, University Hospital of Lausanne, Lausanne, Switzerland; Carter BloodCare & Baylor University Medical Center

**Keywords:** *Mycobacterium tuberculosis*, Xpert MTB/RIF, Xpert MTB/RIF Ultra, acid-fast bacilli, airborne isolation, clinical microbiology, diagnostic microbiology, point-of-care test, smear microscopy, tuberculosis

## Abstract

Xpert MTB/RIF (Xpert) for direct molecular detection of Mycobacterium tuberculosis and rifampin resistance from clinical specimens has dramatically improved the diagnosis of tuberculosis (TB). Xpert MTB/RIF Ultra (Ultra) is proposed as a substitute of Xpert with increased sensitivity and improved rifampin resistance detection. We evaluated the diagnostic performance of Ultra and Xpert for pulmonary TB diagnosis in a low-TB-burden setting. Performance of Ultra and Xpert were compared to culture on respiratory specimens from patients with suspected pulmonary TB (November 2016 to August 2018; *n* = 196) in Lausanne (Switzerland). Clinical data were used to investigate discrepant results. Correlation between semiquantitative result of Ultra and smear microscopy status for the detection of acid-fast bacilli (AFB) was established. The sensitivities of Xpert and Ultra were 82.9% (39/47) and 95.8% (45/47), respectively, when considering all culture-positive specimens, 100% (23/23) for both assays on smear-positive specimens, and 66.7% (16/24) and 91.7% (22/24) on smear-negative specimens. Using culture as gold standard, the specificities of Xpert and Ultra were 97.3% (145/149) and 96.64% (144/149), respectively. All the patients with Ultra-positive results with the new category “trace” were diagnosed with active TB based on clinical findings and microbiological culture. The semiquantitative results of both Xpert and of Ultra positively correlated with the semiquantitative result of AFB detection. Our data support an increased sensitivity of Ultra compared to Xpert in a low-prevalence setting. Correlation between the Ultra semiquantitative result and AFB burden can help in evaluating a patient’s transmission potential.

## Abstract (precis)

Xpert MTB/RIF (Xpert) for direct molecular detection of Mycobacterium tuberculosis and rifampin resistance from clinical specimens has dramatically improved the diagnosis of tuberculosis (TB). Xpert MTB/RIF Ultra (Ultra) is proposed as a substitute of Xpert with increased sensitivity and improved rifampin resistance detection.

## INTRODUCTION

The molecular point-of-care test (POCT) Xpert MTB/RIF (Xpert; Cepheid, Sunnyvale, CA) assay that detects Mycobacterium tuberculosis and resistance to rifampin (RIF-R) has dramatically improved the diagnosis of tuberculosis (TB). Xpert displays reduced turnaround time and shortened patient isolation and time to initiate anti-TB drugs and is more sensitive and specific than smear microscopy for acid-fast bacillus (AFB) detection. This test is now recommended to initiate the microbial diagnosis of tuberculosis (1–3), as well as to address patient’s transmission potential based on the semiquantitative result (1, 4, 5). Despite a very low limit of detection (LOD; ∼131 CFU/ml), Xpert remains less sensitive than liquid culture (LOD ∼1 to 50 CFU/ml) (6). In addition, using Xpert, false-positive RIF-R have been reported, especially in the context of paucibacillary specimens or in case of silent mutation in *rpoB* (7–9). False-negative RIF-R can also be due to mixed population of susceptible and resistant isolates.

The Xpert MTB/RIF Ultra (Ultra; Cepheid) is a new version of the molecular POCT designed to improve the sensitivity for M. tuberculosis DNA detection. In comparison to Xpert, Ultra displays a decreased LOD for M. tuberculosis (∼15.6 CFU/ml); this improvement is achieved by targeting multicopy sequences, namely IS*6110* (∼16 copies/cells) and the IS*1810* (∼5 copies/cells) in Ultra, while Xpert targets the single-copy gene *rpoB* (6, 10). Diagnostic performance established especially in intermediate and high-TB-burden regions reported increased sensitivity of Ultra compared to Xpert (10, 11). In contrast, the specificity of Ultra appeared to be lower than that of Xpert; this was explained by the increased detection of M. tuberculosis DNA from dead bacilli in patients with a history of treated TB (10–13). Indeed, no cross-reactivity with DNA from nontuberculous mycobacteria (NTM) or other bacteria has been reported (10, 11). Rifampicin resistance detection has also been improved in Ultra by relying on the interpretation of the melting curves in the active site of *rpoB* (10, 14). With no decrease in sensitivity compared to Xpert, Ultra can identify with an increased specificity rifampin resistance-associated mutations (14).

In this study, we provide data on the performance and accuracy of Ultra in a region where the prevalence of tuberculosis is low.

## MATERIALS AND METHODS

### Study design.

Our tertiary-care university hospital is located in a low-tuberculosis-prevalence country (Lausanne, Switzerland), with approximately six new cases per year per 100,000 population (Federal Office of Public Health; http://www.bag.admin.ch/). The first collected respiratory sample from each patient with suspected pulmonary tuberculosis, collected from November 2016 to August 2018, were included (*n* = 196; 47 were M. tuberculosis culture positive, and 149 were culture negative). A first pool of specimens (*n* = 69, 34 culture positive and 35 culture negative) consisted of frozen specimens previously tested with Xpert, auramine smear microscopy, and mycobacterial culture. To assess the putative impact of freezing and defrosting frozen specimens, we retested using the Xpert after defrosting and obtained a result similar to that obtained initially (Table S4). A second pool of specimens (*n* = 127, 13 culture positive and 114 culture negative) were tested in parallel with Xpert, Ultra, auramine staining, and mycobacterial culture.

### Microbiology.

All the microbial analyses were performed on the same specimen after it was split for AFB staining, Xpert MTB/RIF (Xpert) analysis, and mycobacterial culture as described earlier (4). When positive, Xpert provides a semiquantitative result, defined by the manufacturer as follows: very low, low, medium, or high. Ultra provides an additional category called “trace.” Smear grading was determined according to the International Union Against Tuberculosis and Lung Disease scale (15).

### Statistics.

Statistics (sensitivity, specificity, and 95% confidence intervals) were calculated using GraphPad Prism 6.00 for Windows (GraphPad Software, La Jolla, CA). Mycobacterial culture was used as the gold standard. The clinical characteristics of patients with discrepant results were reviewed.

### Ethics committee approval.

The study was approved by the local ethics committee (Commission Cantonale d’Ethique de la Recherche sur l’Etre Humain, Lausanne, Switzerland).

## RESULTS

### Comparative performances of Xpert and Ultra.

Compared to culture, the overall sensitivities of AFB detection by Xpert and Ultra were 48.9 (23/47), 82.9% (39/47), and 95.7% (45/47), respectively (Table 1). The sensitivity of Xpert and Ultra with smear-positive specimens was 100% (23/23) for both assays. The sensitivities with smear-negative specimens were 66.7% (16/24) for Xpert and 91.7% (22/24) for Ultra. Among the 47 culture-positive patients, 39 had positive Xpert and positive Ultra specimens. No patients displayed positive Xpert and negative Ultra results, consistent with a noninferiority of Ultra compared to Xpert. In contrast, six patients with culture-positive specimens were positive with the Ultra but negative with Xpert (patients 1 to 6; Table 2 and Table S1). Four of them were Ultra positive “very low,” and two of them were Ultra-positive “trace” (patients 4 and 5). All six specimens were smear negative, and the time to positivity of the culture ranged from 10 to 13 days. Two culture-positive specimens were negative using both Xpert and Ultra (patients 7 and 8). Both specimens were smear negative and were positive after 15 days (Table 2 and Table S1). These data suggest that Ultra-positive/Xpert-negative specimens correspond to paucibacillary specimens. Taken together, these data highlight an increased sensitivity of Ultra compared to Xpert.

**TABLE 1:** Comparative performance of smear microscopy, Xpert MTB/RIF, and Xpert Ultra using culture as the gold standard (*n* = 196 specimens)

Test	*M. tuberculosis* detection			
	% sensitivity (95% CI)			% specificity (95% CI)
	All culture-positive specimens (*n* = 47)	Smear-positive/culture-positive specimens (*n* = 23)	Smear-negative/culture positive specimens (*n* = 24)	All culture-negative specimens (*n* = 149)
Smear microscopy	48.94 (35.28–62.76) 23/47			100 (97.49–100) 149/149
Xpert MTB/RIF	82.98 (69.86–91.11) 39/47	100 (85.69–100) 23/23	66.67 (46.71–82.03) 16/24	97.32 (93.30–98.95) 145/149
Xpert Ultra	95.74 (85.75–99.24) 45/47	100 (85.69–100) 23/23	91.67 (74.15–98.52) 22/24	96.64 (92.39–98.56) 144/149

**TABLE 2:** Clinical characteristics of patients with discrepant results between M. tuberculosis culture, Xpert Ultra, and Xpert MTB/RIF and clinical characteristics of patients with Xpert Ultra positive “trace” results^*a*^

Patient	Sex, age (yr)	Specimen	Smear result	Xpert MTB/RIF	*rpoB* mutation Xpert	Xpert Ultra result	*rpoB* mutation Ultra	MTBC culture result	RIF resistance culture result	Clinical and radiologic finding
1	M, 52	Bronchial aspirate	Negative	Negative	NA	Positive very low	ND	Positive (15 days)	Negative	Cough, lung infiltrate, lymphadenopathy
2	F, 41	Sputum	Negative	Negative	NA	Positive very low	ND	Positive (16 days)	Negative	Cough, wt loss, hemoptysis, fever, miliary lung infiltrate, cavitations, lymphadenopathy
3	F, 43	Sputum	Negative	Negative	NA	Positive very low	ND	Positive (13 days)	Negative	HIV infection, cough, hemoptysis, fever, cavitation
4	M, 17	Bronchial aspirate	Negative	Negative	NA	Positive trace	I	Positive (20 days)	Positive	Cough, lymphadenopathy
5	F, 23	Bronchial aspirate	Negative	Negative	NA	Positive trace	I	Positive (20 days)	Negative	Mediastinal tuberculous lymphadenitis
6	F, 46	Sputum	Negative	Negative	NA	Positive very low	ND	Positive (13 days)	Negative	Hemoptysis, lung infiltrate
7	M, 15	Sputum	Negative	Negative	NA	Negative	NA	Positive (15 days)	Negative	Cough, hemoptysis, cavitation, fever
8	F, 26	Sputum	Negative	Negative	NA	Negative	NA	Positive (15 days)	Negative	Pleural effusion
9	M, 23	Bronchial aspirate	Negative	Positive low	ND	Positive very low	ND	Negative	NA	Cough, cavitation
10	M, 62	Bronchial aspirate	Negative	Positive very low	ND	Positive medium	ND	Negative	NA	Lung infiltrate with cavitation, weight loss; history of TB 20 years before with a relapse 10 years before
11	F, 34	Induced sputum	Negative	Positive very low	ND	Positive very low	ND	Negative	NA	No symptoms; cavitation, M. tuberculosis culture positive on another respiratory specimen
12	M, 39	Bronchoalveolar lavage	Negative	Positive very low	ND	Positive very low	ND	Negative	NA	HIV infection; no symptom; new cavitation and pulmonary nodules; history of TB 7 years before
13	F, 25	Bronchial aspirate	Negative	Negative	NA	Positive trace	I	Negative	NA	Cough, wt loss, asthenia, fever, lymphadenopathy, *M. tuberculosis* PCR and culture positive in a mediastinal cytoponction (EBUS)
14	F, 91	Induced sputum	Negative	Positive very low	ND	Positive trace	I	Positive (12 days)	Negative	Miliary lung infiltrate
15	F, 41	Bronchial aspirate	Negative	Positive very low	ND	Positive trace	I	Positive (18 days)	Negative	History of tuberculosis, new lung infiltrate

a ND, not detected; I, indeterminate; NA, not applicable.

Using culture as gold standard, the specificity of Xpert and Ultra were 97.3% (145/149) and 96.6% (144/149), respectively, with no statistical difference (Table 1). The five patients with positive Ultra and negative culture results received a treatment for active TB based on clinical and radiological findings (3/5; patients 9, 10, and 12) or clinical and radiological findings, together with positive culture on another specimen (2/5; patients 11 and 13).

### Clinical data of patients with Ultra-positive trace results.

The Ultra-positive “trace” is a semiquantitative category that did not exist in the Xpert. All specimens with Ultra-positive trace (patients 4, 5, 13, 14, and 15) were smear negative and Xpert negative, corresponding to paucibacillary specimens. Four patients had culture-positive specimens (patients 4, 5, 14, and 15). One patient had a negative culture on the bronchial aspirate with Ultra-positive trace but was culture positive for M. tuberculosis on another specimen (patient 13) (Table 2). In the tested population, all the specimens with Ultra-positive trace corresponded to patients with active tuberculosis.

### Correlation between the semiquantitative result of Xpert MTB/RIF and Xpert Ultra and smear microscopy results.

We next addressed the correlation between the semiquantitative result of Xpert and Ultra and smear microscopy. Xpert-positive high, medium, and low results corresponded to 100% (3/3), 88.89% (8/9), and 66.67% (6/9), respectively, of smear-positive specimens. Specimens that were Xpert positive very low (*n* = 11) or negative (*n* = 40) were all smear negative. The Ultra-positive high, medium, and low results corresponded to 100% (4/4), 78.57% (10/14), and 40% (2/5), respectively, of smear-positive specimens. Specimens with Ultra-positive very low (*n* = 10), trace (*n* = 5), or negative (*n* = 34) results were all smear negative. These data show that the semiquantitative result of Ultra also correlates with smear examination (Table S2).

### Rifampicin resistance detection.

When considering the 47 culture-positive specimens, all of the isolates were phenotypically susceptible to rifampin. We could not observe any false-positive rifampin resistance prediction either with the Xpert or with the Ultra. We therefore tested specimens (*n* = 3) that did not belong to the studied period and that were phenotypically rifampin positive. Both the Xpert and the Ultra detected the resistance to rifampin in the three specimens (Fig. S3).

## DISCUSSION

Our study aimed to compare the performance and accuracy of Ultra to Xpert in a low-TB-prevalence setting. Ultra detected all Xpert-positive specimens, suggesting a noninferiority of the assay. In addition, Ultra detected M. tuberculosis DNA in five culture-positive/Xpert-negative specimens. These data suggest an increased sensitivity of Ultra compared to Xpert (94.6% versus 81.1% when considering all culture-positive specimens), which confirm results obtained in medium- and high-TB-prevalence countries (88.7% versus 81% in Chakravorty et al. [10] and 88% versus 83% for Dorman et al. [11]). When using culture as the gold standard, Chakravorty et al. and Dorman et al. reported lower specificities for Ultra than for Xpert, i.e., 93 and 96% for Ultra versus 98 and 98.7% for Xpert, respectively (10, 11). This is likely to be the result of the lower detection limit of Ultra and due to the fact that PCR does not discriminate between dead or alive bacteria especially in patients successfully treated for tuberculosis (16, 17). M. tuberculosis DNA can be detected from dead bacilli in specimens from patients treated for tuberculosis; such cases are more likely to occur in medium- and high-TB-prevalence countries (16). Our study also reported culture-negative/Ultra-positive specimens. Using culture as the gold standard, the specificity of Ultra, 96.64%, was lower than that of Xpert, 97.32%, but the difference was marginal and not statistically significant. Interestingly, in our setting all the Ultra-positive “trace” results were considered active TB cases because of an M. tuberculosis-positive culture. Clinical evaluation is paramount for the interpretation of any positive Xpert or Ultra test, with a particular caution when low quantities of DNA are detected.

Finally, a positive correlation of Xpert and Ultra semiquantitative results was found with the smear microscopy results, which could guide airborne isolation measures. This could reduce the time to isolation in emergency services in low-prevalence setting (1, 3, 4). In our institution, we consider patients with negative Ultra results to be likely to be smear negative. In contrast, patients with Ultra-positive high and medium results have a very high probability to be smear positive and correspond to a high transmission potential. Patients with Ultra-positive “low,” “very low,” and “trace” results may correspond to smear-negative patients, which may correspond to a limited, but not negligible, transmission potential. For such patients, a careful analysis of the clinical presentation and the radiologic findings should serve to guide isolation measures. Nevertheless, contact tracing based on threshold cycle (*C_T_*) value or semiquantitative result of molecular tests are still needed to make a direct link between DNA burden and transmission potential. Similar results have been observed in different TB burden settings, suggesting that semiquantitative molecular tests could replace smear microscopy to initiate pulmonary TB diagnosis and treatment as well as to guide airborne isolation strategies (1, 5). Based on these findings, we maintained a smear-independent algorithm based initially on Xpert and now on Ultra to initiate the diagnosis of pulmonary tuberculosis (4).

Regarding rifampin resistance prediction, both Ultra and Xpert correctly detected mutation in *rpoB* linked to the resistance to rifampin, and no false-positive result could be observed. The number of resistance isolates tested was limited due to the low prevalence of multidrug-resistant strains.

In conclusion, our study conducted on clinical specimens collected from patients in a low-TB-prevalence country shows a higher sensitivity of Ultra compared to Xpert. This suggests a potential benefits of Ultra compared to Xpert, which may increase the rate of early case detection. This would improve patient management by the rapid introduction of adequate anti-TB treatment. In addition, early diagnosis improves the control of the spread of the disease by early contact tracing investigation. Cost effectiveness had been addressed for Xpert and might be similar with Ultra; nevertheless, this needs to be addressed in future studies (18, 19). It is paramount to remind that so far a negative Ultra result cannot rule out an active tuberculosis. Finally, as a molecular PCR-based test, Ultra displays a risk of false-positive result due to the detection of DNA from dead tubercles in patients with a history of tuberculosis. This emphasizes the importance of a careful medical history anamnesis and the importance of the pretest probability, as well as the importance of clinical data, for the interpretation of any result, and specifically trace results.

## Supplementary Material

Supplemental file 1
