# Supplementary material for: Added Value of Xpert MTB/RIF Ultra for Diagnosis of Pulmonary Tuberculosis in a Low-Prevalence Setting
Source: J Clin Microbiol. 2019 Jan 30;57(2):e01717-18. doi: 10.1128/JCM.01717-18 (PMC6355522; doi:10.1128/JCM.01717-18)
Supplement: Supplemental file 1 [file 058eb9f2f5360b0c52724f1f9f67c9bf_JCM.01717-18-s0001.pdf]

Table S1. Correlation between Xpert MTB/RIF, Xpert Ultra and culture

Culture	Xpert MTB/RIF	Xpert Ultra	n
Positive	Positive	Positive	39
Positive	Positive	Negative	0
Positive	Negative	Positive	6
Positive	Negative	Negative	2
		Total Culture psoitive	47
Negative	Positive	Positive	4
Negative	Positive	Negative	0
Negative	Negative	Positive	1
Negative	Negative	Negative	32
		Total culture negative	149
		Total	196

Table S2. Correlation between Xpert MTB/RIF, Xpert Ultra semi-quantitative result and smear microscopy results

	Smear microscopy result							
	**Semi-quantitative result**	**NEGATIVE**	**1+**	**2+**	**3+**	**4+**	**Total**	**% of Smear positive specimens Xpert Ultra (Xpert MTB/RIF)**
**Xpert Ultra (Xpert MTB/RIF)**	Positive high			2(1)	2 (1)	1 (1)	5 (3)	100 (100)
	Positive medium	5 (2)	5 (3)	6 (4)	3 (4)	1 (1)	20 (14)	75 (85.7)
	Positive low	4 (5)	2 (4)	1 (4)			7 (13)	42.86 (61.5)
	Positive very low	13 (13)					13	0 (0)
	Positive trace*	5					5	0
	Negative	146					146	0 (0)
	**Total**	**173**	**7**	**9**	**5**	**2**	**196**	

*Xpert Ultra only

Table S3. Rifampicin resistance detection

Specimen	RIFAMPICIN (CULTURE)	XPERT RESULT	XPERT RIFAMPICIN RESISTANCE	ULTRA RESULT	ULTRA RIFAMPICIN RESISTANCE
1	RESISTANT	POSITIVE HIGH	RIF-R DETECTED	POSITIVE HIGH	RIF-R DETECTED
2	RESISTANT	POSITIVE MEDIUM	RIF-R DETECTED	POSITIVE MEDIUM	RIF-R DETECTED
3	RESISTANT	POSITIVE LOW	RIF-R DETECTED	POSITIVE HIGH	RIF-R DETECTED

Table S4. Impact of defrosting

SPECIMEN	INITIAL XPERT RESULT	XPERT AFTER DEFROSTING
SPUTUM	POSITIVE MEDIUM	POSITIVE MEDIUM
BRONCHIAL ASPIRATE	POSITIVE LOW	POSITIVE LOW
BRONCHIAL ASPIRATE	POSITIVE LOW	POSITIVE LOW
